# Feasibility and efficacy of modified fixed citrate concentration protocol using only commercial preparations in critically ill patients: a prospective cohort study with a historical control group

**DOI:** 10.1186/s12871-021-01319-4

**Published:** 2021-03-30

**Authors:** Yan Shi, Han-Yu Qin, Jin-Min Peng, Xiao-Yun Hu, Bin Du

**Affiliations:** Department of medical ICU, Peking Union Medical College Hospital, Peking Union Medical College and Chinese Academy of Medical Sciences, 1 Shuai Fu Yuan, Dongcheng District, Beijing, 100730 China

**Keywords:** Regional citrate anticoagulation (RCA), Continuous veno-venous hemofiltration (CVVH), Commercial preparations, Protocol, Filter lifespan, Convenience, Critically ill patients

## Abstract

**Background:**

The cumbersome program and the shortage of commercial solution hindered the regular application of regional citrate anticoagulation (RCA). It is urgent to simplify the protocol using only commercial preparations. The aim of this study was to explore the feasibility and efficacy of the modified protocol for continuous veno-venous hemofiltration (CVVH) in unselected critically ill patients.

**Methods:**

A prospective cohort study was conducted in 66 patients who received a new protocol combining fixed citrate concentration with modified algorithm for supplements (i.e., fixed protocol), and compared the efficacy, safety and convenience for this group to a historical control group with a traditional protocol (*n* = 64), where citrate was titrated according to the circuit ionized calcium concentration (i.e., titrated protocol). The convenience was defined as the demand for monitoring test and dose adjustment of any supplement.

**Results:**

The filter lifespan was 63.2 ± 16.1 h in the fixed group and 51.9 ± 17.7 h in the titrated group, respectively. Kaplan-Meier survival analysis demonstrated longer circuit lifetime for fixed group (log-rank, *p* = 0.026). The incidence of circuit clotting was lower in the fixed protocol (15.2% vs. 29.7% in the titrated protocol, *p* = 0.047). Moreover, compared with the titrated group, patients with fixed protocol had less demand for monitoring test and dose adjustment of any supplement (the number of times per person per day) (3.3 [IQR 2.3–4.5] vs. 5.7 [IQR 3.3–6.9], *p* = 0.001 and 1.9 [IQR 0.5–2.7] vs. 6.3 [IQR 4.2–7.9], *p* < 0.001; respectively). No new onset bleeding complications occurred in all patients. The overall incidence of suspected citrate accumulation was 4.6% and there was no difference between the two groups (*p* = 0.969), yet a lower rate of metabolic alkalosis was found in the fixed group (3.0% vs. 14.1%, *p* = 0.024).

**Conclusions:**

Our modified fixed citrate concentration protocol is feasible, safe and effective to enhance the circuit lifespan and the convenience of implementation while maintaining a similar safety when compared to the traditional protocol. Using only commercial preparations may be helpful for widespread application of RCA.

**Trial registration:**

Clinicaltrials.gov. NCT02663960. Registered 26 January 2016.

**Supplementary Information:**

The online version contains supplementary material available at 10.1186/s12871-021-01319-4.

## Background

Continuous renal replacement therapy (CRRT) is an important tool in the care of critically ill patients, and adequate anticoagulation is needed to prevent circuit clotting. Kidney Disease Improved Global Outcomes (KDIGO) guideline suggests regional citrate anticoagulation (RCA) as the first-line method in patients without contraindication for citrate [1]. However, in clinical practice, the use of RCA is still limited because of concerns related to the risk of metabolic complications and the complexity of the proposed protocols [2–5]. In particular, a shortage of commercial solution and high costs also hinder its regular application in developing countries. For example, in China, clinicians prefer heparin to RCA for continuous veno-venous hemofiltration (CVVH), and the major factors limiting the use of citrate are high cost (32%), complex program (21%), limited source of commercial solution (18%) and concerns of complications (12%) [6]. So, it is urgent to simplify the protocol using only commercial preparations.

Nowadays, the most frequently used method is a separate citrate infusion combined with pre or post-dilution replacement fluid (RF) [7–12]. This scheme needs to adjust at least three solutions according to the mechanism of citrate anticoagulation and its metabolic [2–5]: (1) the amount of citrate needs to be titrated according to the circuit ionized calcium (c-iCa) levels; (2) a systemic calcium infusion replaces the calcium loss in the effluent and (3) citrate as a buffer substrate may require adjustment of bicarbonate supplement. Also, strict laboratory monitoring is needs to prevent serious metabolic complications. Therefore, the traditional scheme increases complexity, labor intensive and iatrogenic errors.

More recently, RCA protocol has been simplified by using citrate-buffered RF in predilution mode, which is isotonic and acts as an anticoagulant and buffer [13–20]. However, since CVVH dose was coupled to citrate dose, the inflexibility in flow rates changes and insufficient dialysis dose were its main disadvantages [3, 5, 21]. In addition, the assumption of using citrate-buffered solution instead of additional buffer infusion was limited by variable alkali load provided by citrate in the different CRRT mode, which was different between amount of citrate infusion and loss into the effluent (e.g, CVVH, continuous veno venous hemodialysis [CVVHD] or continuous veno venous hemodiafiltration [CVVHDF]) [2, 5, 22, 23], in clinical practice, it was not sufficient to compensate for acidosis, so an additional infusion of bicarbonate was required [16–18]. Also, since anticoagulation was coupled with metabolic control, the buffer cannot be titrated separately and limited the ability to fine tune the control of metabolic disturbances [5, 21, 22]. Besides, the citrate-buffered solution has to be custom-made, increasing cost and labour. Although a dilute citrate solution has recently become available in Europe known as prismocitrate 10/2, anticoagulant citrate dextrose (ACD) solution and bicarbonate buffered fluid are the common used in most countries. Moreover, most medical centers in China have to use calcium-containing RF due to an absence of commercially available calcium-free solution [6, 9, 10].

In order to widely employ RCA, we devised a new protocol using fixed circuit citrate concentration combined with modified algorithm for supplements (i.e., fixed protocol) for CVVH in unselected adult critically ill patients, and compared the efficacy, safety and convenience for this group to a historical control group with a traditional titrated protocol.

## Methods

### Study design and selection of patients and controls

This prospective study was conducted at a 15-bed medical intensive care unit (ICU) of the Peking Union Medical College Hospital, Beijing, China. After 2 months of staff training, from August 2015, our institution began to employ a new protocol as part of the routine method for CVVH.

We prospectively recruited patients older than 18 years of age who required RCA-CVVH. Patients were excluded if they:1) required systemic heparin anticoagulation (SHA), because RCA will no longer be adopted according to our usual practice. On the contrary, the subcutaneous injection of low molecular weight heparin (LMWH), as a routine prophylactic or therapeutic method for deep venous thrombosis in patients with low risk of bleeding, does not affect the choice of anticoagulant method for CVVH and the recruitment of patients; 2) had incomplete data; 3) had CVVH running time less than 6 h. Historical control group of patients who met the same criteria have been screened backward since August 2015.

This study was approved by the institution’s Research Ethics Board (No. ZS-950), and written informed consent was obtained from all prospective study participants or their relatives.

### RCA-CVVH protocols

Before and after implementation of a new protocol, predilution CVVH was performed using same commercially anticoagulant citrate dextrose solution -A (ACD-A) (Na^+^ 224, citrate 113, bicarbonate 203 all in mmol/L, Nigale Biotechnology Co. Ltd., Sichuan, China) and calcium- containing alkali-free RF (Na ^+^ 113, Cl ^−^ 118, Mg ^2+^ 0.797, Ca ^2+^ 1.60 all in mmol/ L; Qingshan Likang, Pharmaceutical Co. Ltd., Chengdu, China). The same standards were adopted, such as CRRT devices (Aquarius or Diapact), catheter type (a double-lumen 12-F catheter, Arrow International Inc., USA), vascular access (placed in the internal jugular or femoral vein) and haemofilter (DIACAP Acute L, 2.0 m^2^, B. Braun Melsungen AG, Germany). The circuit was run for 72 h unless there was filter clotting, transmembranous pressure exceeded 300 mmHg, transportation was required, or the predefined clinical target was reached. The separate ACD-A was administered in the “arterial” line of the extracorporeal circuit. The flow rate of RF was flexibly adjusted to achieve a prescribed dialysis dose of at least 25 ml/kg/h. Calcium gluconate (10%) and sodium bicarbonate solutions (5%) were infused through the return line of the circuit.

For the titrated protocol, ACD-A was initiated with 2.5% of blood flow, corresponding to 3 mmol/L of extracorporeal blood (eg, blood flow is typically 7200 ml/h [120 ml/min] and ACD-A is run at 180 ml/h) [8, 9, 20]. Then, the citrate flow was adjusted to maintain the c-iCa of 0.2 to 0.4 mmol/L, as described in previous studies [8, 9, 19, 20]. The initial calcium substitution flow was set at 6.1% of ACD-A flow and titrated to maintain the systemic ionized calcium (s-iCa) of 1.0 to 1.2 mmol/L (see Additional file 1). The initial infusion rate of sodium bicarbonate was set at 5% of RF flow (equivalent to HCO3^−^ concentration of 30 mmol/L in the RF), and then was adjusted to keep blood pH within the normal range.

The fixed protocol has been modified in three aspects: First, considering predilution calcium- containing solution increased calcium load in vitro (e.g., the calcium load increased by 20–30% when the RF flow rate was usually set at 2–2.5 l/h) [5, 9], we determined a target citrate concentration of 4 mmol/L instead of the lowest concentration reported of 3 mmol/L [8, 9, 20, 23]. ACD-A infusion dose corresponding to blood flow was shown in Additional file 2. This constant concentration was maintained during CRRT unless citrate accumulation (CA) was suspected, which were resolved by reducing either blood flow or circuit citrate concentration until discontinuation. Second, calcium substitution flow was determined through a roughly estimate the amount of calcium loss at 0.8 mmol per liter total effluent flow based on the preliminary experiment results involving 10 patients (see Additional file 3). For patients with hypocalcemia at start CVVH, a bolus dose of calcium gluconate was delivered (0.3 ml/kg). Third, bicarbonate substitution flow was set at 3.5% of RF flow (corresponding to HCO3^−^ of 20 mmol/L in the RF), which was determined by using a mathematical model to roughly estimate the alkali load provided by citrate (i.e., citrate metabolic load (mmol/h) = [citrate solution concentration (mmol/L) x citrate flow rate (l/h)] – [effluent rate (l/h) x estimated citrate blood concentration (mmol/L) x sieving coefficients of citrate]) [20]. If necessary, the infusion rate of calcium or bicarbonate solution should also be adjusted to hold s-iCa and pH within the normal range.

The same monitoring algorithm was used in the two groups. The first measurements of c-iCa, s-iCa, pH and HCO3^−^ levels were done 1 h after initiation of CVVH and every 4 to 6 h during the first 24 h. Then these measurements were done according to clinical needs. Moreover, c-iCa level was measured 1 h after any change in blood flow, citrate, RF or calcium flow rate. The liver and kidney functions, haemoglobin, platelet counts, total serum calcium, prothrombin time and activated partial thromboplastin time were measured at least daily.

### Data collection

All data were collected through the hospital information system and the prescriptions for CVVH, and included patient’s age, sex, comorbidity, severity score using the acute physiology and chronic health evaluation (APACHE) II and the sequential organ failure assessment (SOFA) score, application of LMWH, physiological support, reasons for start and stopping CVVH, filter lifetime, CVVH settings (e.g., blood flow, ACD-A flow, RF flow and effluent flow), frequency of dose adjustment for any supplement, laboratory variables and monitoring requirements during CVVH and ICU mortality at 28 days.

### Study endpoints and definition

The primary endpoint was filter lifespan of the first circuit. The reasons for stopping CVVH were divided elective discontinuation (e.g., replacement per protocol at 72 h, clinical target achievement, CA, death or discharge) or spontaneous circuit failure (circuit clotting, transmembrane pressure > 300 mmHg, catheter malfunction) [12, 14]. The secondary endpoint was the convenience, which was defined as the demand for monitoring tests and dose adjustment of any supplement per person per day. We also compared the incidence of complications, including severe metabolic disorders (defined as metabolic alkalosis with pH > 7.5, metabolic acidosis with pH < 7.25, hypocalcemia with s- iCa < 0.7 mmol/L, hypercalcemia with total serum calcium ≥2.75 mmol/L, hypernatremia with Na ≥ 150 mmol/L), bleeding disorders (defined as the decline of hemoglobin 10 g/L within 12 h) and suspected CA (identified as a total calcium/ionic calcium ratio ≥ 2.5), as described *in the literature* [17, 19, 20].

### Sample size and statistics

We hypothesized a difference of 10 h in mean circuit survival for different RCA protocol, with an estimated mean circuit survival of 40 h and pooled standard deviation of 20 h, which was reported by some randomized clinical trial and our experience [12, 17, 19]. To obtain 80% power with a two-sided α level < 0.05, the sample size was targeted at 63 patients per group and final sample size at 70 patients per group is needed to correct for an expected 10% drop out.

Continuous variables were presented as means and standard deviation (SD) for normally distributed data, and medians and interquartile range (IQR) for all other data, whereas categorical variables were presented as number (percentage). Continuous variables were compared with the use of the Student’s t test or Mann-Whitney test, while categorical variables were compared using chi-square test or Fisher’s exact test. The Kaplan-Meier survival curve was used to depict the cumulative survival, and a log-rank test was used to assess the difference. All comparisons were unpaired, and all tests of significance were two-tailed. A *p* value < 0.05 was considered statistically significant. The statistical analyses were performed with SPSS statistics software (version 20.0; SPSS Inc., Chicago, IL).

## Result

### Clinical characteristics and CVVH implementation

We consecutively screened 140 patients forward (until May 2016) and backward (until September 2014). Four patients (one of requiring SHA and 3 of running time < 6 h) in a prospective session and 6 patients (2 of insufficient data and 4 of running time < 6 h) in a retrospective phase were excluded, and 130 patients were included in the final analysis (64 in the titrated group and 66 in the fixed group).

The mean age was 63.1 ± 15.9 years old and the median body weight was 70.7 kg. Twenty-five patients had a history of chronic kidney dysfunction (stage 3 or greater according to KDIGO) and 8 had dialysis-dependent; Two patients had liver dysfunction with Child-Pugh B or C. The most common reason for CVVH was due to sepsis-induced acute kidney injury, accounting for 83.8%. Forty-three patients (33.1%) received subcutaneous LMWH treatment. The APACHE II score was 22.3 ± 4.7. One hundred and 18 patients (90.8%) required mechanical ventilation, 115 patients (88.5%) received vasopressors. ICU mortality rate at 28 days was 32.3% (*n* = 42). Demographic, clinical characteristics and laboratory examination at baseline were well-matched across groups (Table 1).

**Table 1:** The patients’ characteristics and laboratory parameters at CVVH start between the two groups

Variables	Fixed group (*n* = 66)	Titrated group (*n* = 64)	*P* value
Age, years, mean (SD)	62.7 (18.4)	64.1 (17.6)	0.379
Female gender, n (%)	24 (36.4)	22 (34.4)	0.813
Body weight, kg, median (IQR)	69.2 (61.5–74.3)	70.5 (63.5–74.5)	0.852
Comorbidities, n (%)			
Chronic kidney disease ^a^	13 (19.7)	12 (18.8)	0.891
Dialysis-dependent	6 (9.1)	2 (3.1)	0.157
Diabetes mellitus	14 (21.2)	17(26.6)	0.474
Hypertension	19 (28.8)	16 (25.0)	0.626
Liver dysfunction ^b^	1 (1.5)	1 (1.6)	0.983
Cardiac insufficiency ^c^	3 (4.5)	3 (4.7)	0.969
Severity of illness			
APACHE II score, mean (SD)	23.1 (6.8)	22.7 (6.4)	0.541
SOFA score, mean (SD)	8.0 (2.7)	8.3 (2.3)	0.672
Vasopressors, n (%)	58 (87.9)	57 (89.1)	0.833
Mechanical ventilation, n (%)	60 (90.9)	58 (90.6)	0.955
Indication for CVVH, n (%)			
Sepsis-induced AKI	54 (81.8)	55 (85.9)	0.523
Fluid overload	5 (7.6)	5 (7.8)	0.960
Tumorlysis syndrome	4 (6.1)	2 (3.1)	0.425
Others	3 (4.5)	2 (3.1)	0.674
Application of LMWH, n (%)	19(28.8)	24(37.5)	0.352
Laboratory parameters			
Hemoglobin, g/L, mean (SD)	89.1 (19.2)	85.5 (19.7)	0.573
Platelet count, 10^9^/L, median (IQR)	74.1 (50.7–97.5)	81.5 (60.8–102.2)	0.153
APTT, S, mean (SD)	42.6 (8.7)	43.2 (8.1)	0.754
PT, S, mean (SD)	14.0 (2.7)	14.6 (2.3)	0.805
Serum albumin, g/L, mean (SD)	23.3 (2.4)	24.7 (3.5)	0.672
Bilirubin, mmol/l, mean (SD)	29.4 (7.5)	27.0 (10.8)	0.718
ALT, U/L, mean (SD)	68 (28.8)	56 (25.5)	0.182
Creatinine, μmol/l, median (IQR)	229 (169–559)	240 (143–497)	0.756
Urea, mmol/l, median (IQR)	33.9 (15.6)	30.8 (14.7)	0.347
Lactate, mmol/l, median (IQR)	4.8 (2.7–6.8)	4.5 (3.1–6.3)	0.498
ICU death at 28 days, n (%)	20 (30.3)	22 (34.4)	0.239

*Abbreviations: AKI* acute renal injury, *ALT* Alanine Aminotransferease, *APACHE*, acute physiology and chronic health evaluation, *APTT* activated partial thromboplastin time, *CVVH* continuous veno-venous hemofiltration, *ICU* Intensive Care Unit; *IQR* interquartile range, *LMWH* low molecular weight heparin, *PT* prothrombin time, *RCA* regional citrate anticoagulation, *SD* standard deviation, *SOFA* Sequential Organ Failure Assessment

^a^ Chronic kidney disease defined as stage 3–5 according to Kidney Disease Improving Global outcome in 2012;

^b^ liver dysfunction defined as Child-pugh B-C stage

^c^ Chronic cardiac insufficiency defined of stage III-IV according to New York Heart Association

There was no significant difference between the two groups in terms of blood flow, RF and total effluent flow rate (Table 2). As expected by the protocol, a significant decrease in the initial infusion rate (ml/h) of calcium and bicarbonate was observed in the fixed group than that in the titrated group (9.5 vs.12.1, *p* = 0.038 and 70 vs.105, *p* = 0.015; respectively). Also, the initial ACD-A flow (ml/h) of the fixed group was slightly higher than that of the titrated group (261 ± 13 vs 191 ± 11, *p* = 0.037), but was similar at the end of CVVH (259 ± 13 vs. 242 ± 15, *p* = 0.378). Serum creatinine, blood urea nitrogen and potassium levels were significantly decreased with CVVH therapy and there were similar between the two groups (Table 2).

**Table 2:** CVVH setting, acid-base and metabolic control at CVVH start and end between groups

Variables	Fixed group		Titrated group	
	Start	End ^a^	Start	End ^a^
CVVH settings, mean (SD)				
Blood flow (ml/min)	122 (7)	122 (6)	123 (10)	122 (8)
Citrate flow (ml/h)	261 (13) ^*^	259 (13)	191(11)	242 (15)
Replacement flow (ml/h)	2148 (102)	2218 (175)	2292 (122)	2198 (103)
Total effluent flow (ml/h)	2743 (270)	2612(347)	2698 (470)	2745 (229)
Sodium bicarbonate (ml/h)	70 (10) ^*^	60 (25)	105 (25)	63 (23) ^**^
Calcium gluconate (ml/h)	9.5 (1.2) ^*^	8.3 (2.7)	12.1 (1.2)	8.1 (3.4)
Acid-base status				
pH, mean (SD)	7.29 (0.1) ^**^	7.39 (0.1)	7.26 (0.1) ^**^	7.42 (0.2)
Bicarbonate, mean (SD)	19.3 (4.1) ^**^	25.5 (3.5) ^*^	18.7 (3.5) ^**^	27.9 (4.2)
Base Excess, median (IQR)	−6.0 (−6.5, −5.4) ^**^	1.7 (−0.5, 2.9) ^*^	−5.7 (−6.7,− 5.3) ^**^	4.1(− 0.7,4.7)
Metabolic control				
Serum creatinine, μmol/L	229 (169–559) ^**^	136 (85–178)	240 (143–497) ^**^	148 (93–185)
Blood urea nitrogen, mmol/L	33.9 (15.6) ^**^	22.1(9.8)	30.8 (14.7) ^**^	20.3(6.7)
Sodium, mmol/L	138.7 (3.5)	140.1 (5.7)	138.5(5.8)	141.0(5.2)
Potassium, mmol/L	5.4 (1.8)^**^	3.9 (1.3)	5.3 (1.5) ^**^	3.9 (1.3)
Total serum calcium, mmol/L	2.12 (0.3)	2.10 (0.2)	2.13 (0.2)	2.12 (0.3)
Ionized calcium, mmol/L	1.07 (0.15)	1.05 (0.14)	1.08 (0.13)	1.06 (0.08)

*Abbreviations: CVVH* continuous veno-venous hemofiltration, *IQR* interquartile range, *SD* standard deviation

^*^: *p* < 0.05 for fixed vs titrated group; ^**^: *p* < 0.05 for CVVH start vs end in the each group

^a^: The data at the end of CVVH comes from the average of the day

### Filter lifetime and c-iCa monitoring parameters

**Fig. 1:**
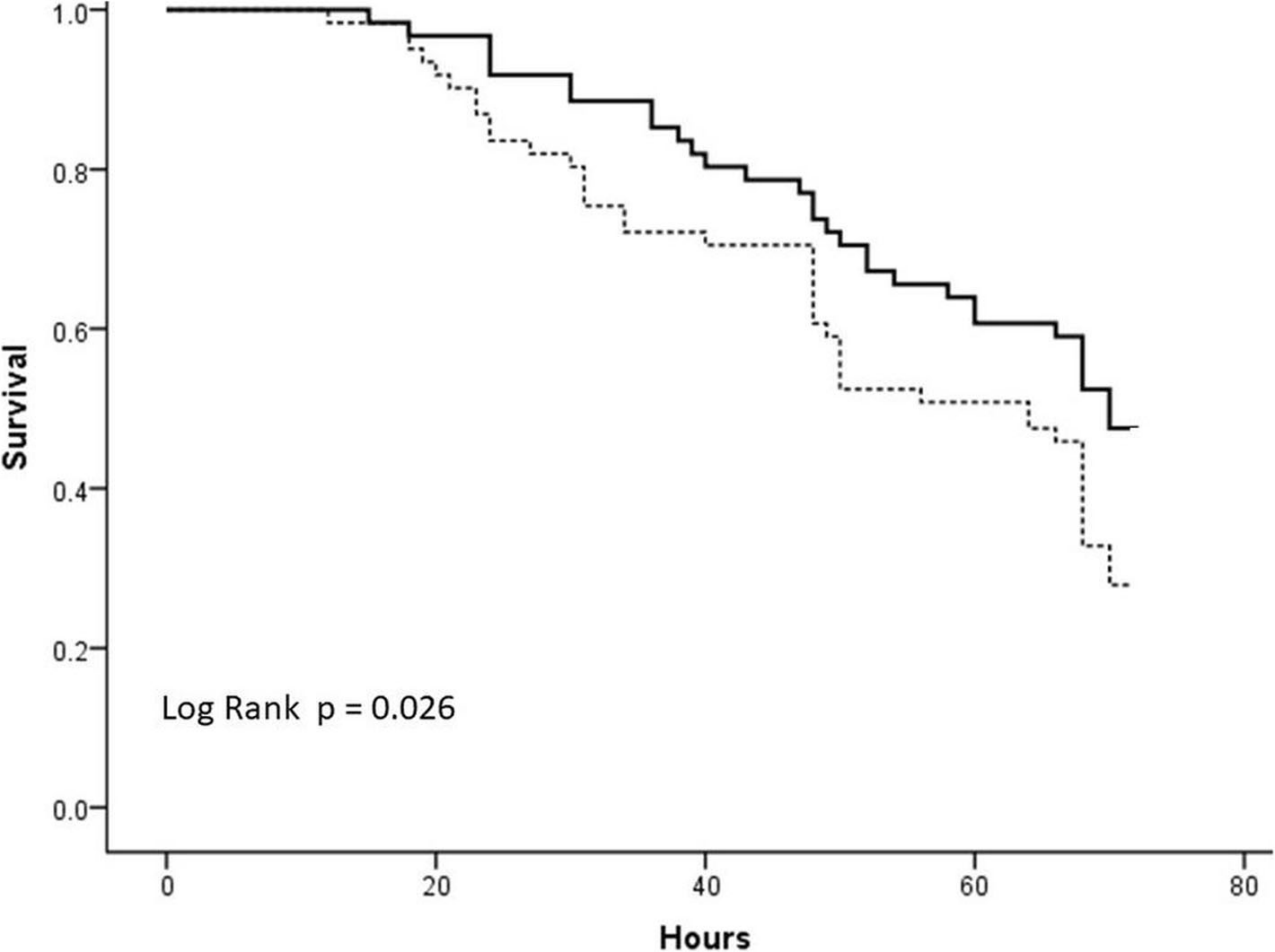
The Kaplan-Meier circuit survival curve according to diffrent citrate anticoagulation protocol. Survival curves derived from the analysis of CVVH termination for any cause. Continuous line represents fixed protocol, dotted line represents titrated protocol

The total running time of the fixed group and the titrated group was 3266 h and 4336 h, respectively. The circuit lifetime was 63.2 ± 16.1 h with fixed protocol and 51.9 ± 17.7 h with titrated protocol (*p* = 0.014). Ninety-one percent, 85 and 68% of the hemofilters with fixed protocol still operated at 24, 48, and 72 h, respectively, while 81, 69 and 53% in the titrated group, respectively. The Kaplan-Meier survival analysis demonstrated longer circuit lifetime for fixed group (log-rank, *p* = 0.026) (Fig. 1). The rate of circuit failure due to clotting in the fixed group was lower than that in the titrated group (15.2% vs 29.7%, *p* = 0.047), but there was no difference in the termination rate due to other reasons (Table 3). Even for the clotted filters, the circuit lifespans were significantly prolonged by the fixed protocol, when compared to the titrated scheme (48.7 ± 15.4 h vs 36.5 ± 18.4 h, *p* = 0.044).

**Table 3:** Reasons for stopping continuous veno-venous hemofiltration

	Fixed group	Titrated group	*P* value
Spontaneous circuit failure, n (%)			
Clotting	10 (15.2)	19 (29.7)	0.047
Catheter dysfunction	3 (4.5)	4 (6.3)	0.667
Elective discontinuation, n (%)			
Replaced as planned after 72 h	45 (68.2)	34 (53.1)	0.079
Clinical target achievement	4 (6.1)	3 (4.7)	0.729
Death	3 (4.5)	2 (3.1)	0.674
Citrate Accumulation	1 (1.5)	2 (3.1)	0.541

During CRRT, the c-iCa levels in 525 and 714 blood samples of the fixed group and the titrated group were measured respectively. The first measured concentration of c-iCa was 0.33 ± 0.05 mmol/L in the fixed group, 98.5% of which was within the target range of 0.2–0.4 mmol/L, while that was 0.38 ± 0.09 mmol/L and 90.6% in the titrated group, respectively (*p* < 0.001 and *p* = 0.031, respectively). Of all measured c-iCa, 1.5% exceeded 0.4 mmol/L, 10.6% had c-iCa values at 0.35 to 0.4 mmol/L, 84.8% was at 0.2 to 0.34 mmol/L and 3.1% was less than 0.2 mmol/L in the fixed group, while 7.7, 46.9, 42.5 and 2.9% were found in the titrated group, respectively (*p* < 0.001). Also, the medium c-iCa levels (mmol/L) in the fixed protocol were lower than that in the titrated protocol (0.31 [IQR 0.25–0.36] vs 0.37 [IQR 0.29–0.39], *p* = 0.002).

### Convenience of implementation

During CVVH, more frequent adjustment (the number of times per person, per day) of any infusion pump was found in the titrated group (6.3 [IQR 4.2–7.9] vs. 1.9 [IQR 0.5–2.7], p < 0.001). Among them, the adjustment frequency of calcium and bicarbonate infusion pump in the titrated group versus the fixed group were 2.5 (IQR 2.0–4.1) vs. 0.5 (IQR 0.3–0.9) and 3.5 (IQR 2.5–4.8) vs. 1.5 (IQR 0.8–2.7), respectively (all *p* < 0.05). In particular, 87.5% of patients with titrated protocol needed to adjust the dosages of ACD-A infusion pump, with a change of 28.5 (IQR 12.8–43.5) ml/h, but only two patients suspected of CA were adjusted in the fixed group. In addition, 703 and 1096 blood samples were taken for monitoring tests during CRRT in the fixed group and the titrated group, respectively, and patients with fixed protocol had less monitoring needs (3.3 [IQR 2.3–4.5] vs. 5.7 [IQR 3.3–6.9], *p* = 0.001).

### Complications

No new onset bleeding complication attributable to RCA was observed. In terms of metabolic complications, hypernatremia (5.4%, *n* = 7) and metabolic alkalosis (8.5%, *n* = 11) were common in all patients. The incidence of hypernatremia was comparable between groups (6.1% in the fixd group vs 4.7% in the titrated group, *p* = 0.667), while the incidence of metabolic alkalosis was higher in the titrated group (14.1% vs 3.0%, *p* = 0.024). However, all patients had normalization of serum bicarbonate levels after reducing or discontinuation of alkali infusion. The s-iCa levels (mmol/L) were relatively stable and there was no difference between groups (1.02 ± 0.17 in the fixed group vs. 1.04 ± 0.14 in the titrated group, *p* = 0.299). Aside from those who presented with hypocalcemia before CVVH, only two patients (one in each group, *p* = 0.983) presented with asymptomatic hypocalcemia. Moreover**,** six patients (3 cases in each group, *p* = 0.969) were suspected of CA, and three of them died from refractory shock, the remaining patients were corrected after decreasing the dose of citrate infusion.

## Discussion

The cumbersome program and the shortage of commercial solution hindered the regular application of RCA. In this study, we presented a new protocol using fixed circuit citrate concentration combined with modified algorithm for calcium and buffer supplement, and demonstrated that it improved the circuit lifespan and the convenience while maintaining a similar safety, when compared with the titrated protocol. Using only commercial preparations may contribute to its widespread application.

Our new protocol has different features from the reported constant citrate concentration schemes, which used either a fixed flow rate of blood and citrate-buffered solution [13–16] or a constant ratio between them [17–20]. The obvious disadvantages were not only that citrate- buffered solution was not generally commercially available, but also that the adjustment of flow rate was inflexible for the former, and the fixed relationship between citrate and blood flow was uncertain for the latter because RF rate varied with ultrafiltration flow and desired fluid removal [2, 5]. By contrast, the separate citric infusion overcame these shortcomings, as the anticoagulation effect was not coupled to solute control. Also, the programs published so far have required calcium-free solutions, and when calcium-containing solution as only option, the appropriate citrate concentration was not clear due to an increased citrate load and accumulation risk [3, 5, 14]. Therefore, the present study also explored this issue.

The shorter filter lifespan in the titrated group was likely due to the lower initial citrate concentration (almost 3 mmol/L of extracorporeal blood), which resulted in a high initial c-iCa level, and the consequent frequent modification of citrate or calcium supplement flow might affect the stability of c-iCa levels (e.g., about 10% of all measurements wasn’t achieved the target range of 0.2–0.4 mmol/L) [4, 5, 24]. On the contrary, target c-iCa was easily achieved without further change of citrate flow rate in the fixed group. Therefore, appropriate circuit citrate concentration was prerequisite for maintaining stable target c-iCa levels, which was also associated with an adequate circuit lifetime. Moreover, recently published studies suggested that the best effect of anticoagulation occurred when the c-iCa was < 0.35 mmol/L, there was near total inhibition of coagulation and was better than the usual target (≤ 0.4 mmol/L) [3, 5, 25, 26]. In the present study, the median c-iCa level and most of the c-iCa levels measured during CVVH could be maintained at the optimal concentration (i.e., < 0.35 mmol/L) in the fixed group, which may partly explain the difference of filter survival between groups. In addition, the circuit duration in our study was longer or similar to those described by others, which reported filter lifespan of 26–70 h using calcium-free RF and citrate concentration of 3–5 mmol/L [8–20]. Because the filter survival was affected by many factors, such as catheter size, venous access, blood flow rate and ultrafiltration rates, it was difficult to compare the reasons for the differences between different studies, but it was worth mentioning that the application of LMWH in one-third of our patients (but no difference between groups) may be associated with longer filter lifetime. The latest research by Giani reported that RCA may prove useful also in patients treated with systemic heparinization, as it maximise circuit patency and reduces circuit clotting rate [27]. Of course, the reason for stopping CVVH, such as achievement of clinical target, filter replacement as scheduled or until clotting may also affect the filter lifetime, nevertheless, the rate of circuit clotting in our study was somewhat lower than the reported range of 16–46% [12–14, 16–18]. Besides, the incidence of suspected CA was 4.6%, which was comparable with the rate of 3–9% in other studies [14–17, 20]. These results demonstrated that the circuit citrate concentration of 4 mmol/L was effective and safe for predilution calcium-containing solution.

Our study was the first to compare the convenience of different RCA protocol, and found that the fixed protocol was more convenient in terms of demand reduction for monitoring test and dose adjustment of any supplements. This was mainly due to the fact that the algorithm for calcium and bicarbonate supplements has been improved to replace the traditional on-demand supplementary methods. While the modification of formula was attributed the constant citrate concentrations, which made it possible to use the model to roughly estimate citrate metabolic load, buffers balance and effluent calcium loss [3–5, 20, 24]. Although calcium supplement was very complex, our algorithm was in concordance with some studies, where the calcium infusion rate can be determined according to the extracorporeal calcium loss [28, 29]. Also, the incidence of metabolic alkalosis was reduced by adjusting the HCO3^−^ concentration in the RF. Meanwhile, more effective controls of acid-base status and calcium balance also reduced the need for monitoring test. Particularly, regular measurement of c-iCa may be omitted due to almost universally below 0.4 mmol/l in the fixed protocol, which may have potential benefits in reducing costs and nurse workload.

We also found that the overall metabolic complications were rather low and easily controlled. On the concerns of CA, in general, shock was claimed to be the risk factor for CA and a contraindication for RCA [1, 12, 30]. Indeed, clinical practice showed that a considerable proportion of patients with shock could tolerate citrate anticoagulation, especially those if circulation improved [31, 32]. Our results also indicated that the overall incidence of CA even in a significant number of patients with shock or hypoxemia (account for about 90%) was low.

Several limitations should also be noted. First, due to its historical control group, a longer filter lifespan with fixed protocol may reflect an improvement in the standard of care, but using same CRRT criteria may help to minimize bias. Second, the blood flow rate was usually set between 120 and 150 ml/min in our institution and further research is needed to evaluate the safety in high blood flow setting, which corresponds to high citrate dose. Third, few patients with severe liver disease were enrolled, who were the most relevant patient group at risk to develop CA, our results must be applied with caution to these patients. Fourth, calcium-containing solution is far from optimal for RCA, however, in case of calcium-free replacement solution shortage, it may be our only option. Lastly, the metabolic load of citrate and calcium homeostasis varied in different CRRT modes, therefore, other modes (e.g., post-dilution CVVH, CVVHD, CVVHDF) needs to be evaluated in future research.

## Conclusions

Our modified fixed citrate concentration protocol seems a feasible, safe, and effective to improve the circuit lifespan and convenience while maintaining a low incidence of metabolic complications when compared to the titrated protocol. Using only commercial preparations that were available in most countries may represent a significant step toward widespread acceptance of RCA.

## Supplementary Information

**Additional file 1.**

**Additional file 2.**

**Additional file 3.**

## Data Availability

The datasets during and/or analysed during the current study available from the corresponding author on reasonable request.

## Abbreviations

ACD-A: anticoagulant citrate dextrose solution-A
APACHE: Acute physiology and chronic health evaluation
CA: Citrate accumulation
CRRT: Continuous renal replacement therapy
CVVH: Continuous veno-venous hemofiltration
CVVHD: Continuous veno venous hemodialysis
CVVHDF: Continuous veno venous hemodiafiltration
c-iCa: Circuit ionized calcium
ICU: Intensive care unit
IQR: Interquartile range
KDIGO: Kidney Disease Improved Global Outcomes
LMWH: Low molecular weight heparin
RCA: Regional citrate anticoagulation
RF: Replacement fluid
s-iCa: Systemic ionized calcium
SHA: Systemic heparin anticoagulation
SOFA: Sequential organ failure assessment
