# Supplementary material for: Feasibility and efficacy of modified fixed citrate concentration protocol using only commercial preparations in critically ill patients: a prospective cohort study with a historical control group
Source: BMC Anesthesiol. 2021 Mar 30;21:96. doi: 10.1186/s12871-021-01319-4 (PMC8008573; doi:10.1186/s12871-021-01319-4)
Supplement: Supplementary file 1 — Additional file 1. [file 12871_2021_1319_MOESM1_ESM.docx]

**Additional file 1.** Adjustment scheme of ACD-A and 10% calcium gluconate solution in the titrated group

| c-iCa (mmol/L) | ACD-A (ml/h) | s-iCa (mmol/L) | Calcium gluconate (ml/h) |
| --- | --- | --- | --- |
| < 0.20 | Reduction by 5 | > 1.45 | Reduction by 6 |
| 0.20 – 0.40 | No change | 1.21 – 1.45 | Reduction by 3 |
| 0.41 – 0.50 | Increase by 5 | 1.00 – 1.20 | No change |
| > 0.50 | Increase by 10 | 0.9 – 1.00 | Increase by 3 |
|  |  | < 0.90 | increase by 6 |

Abbreviations: ACD-A: anticoagulant citrate dextrose solution-A; c-iCa: circuit ionized calcium concentration; s-iCa: systermt ionized calcium concentration
