# Supplementary material for: Feasibility and efficacy of modified fixed citrate concentration protocol using only commercial preparations in critically ill patients: a prospective cohort study with a historical control group
Source: BMC Anesthesiol. 2021 Mar 30;21:96. doi: 10.1186/s12871-021-01319-4 (PMC8008573; doi:10.1186/s12871-021-01319-4)
Supplement: Supplementary file 2 — Additional file 2. [file 12871_2021_1319_MOESM2_ESM.docx]

**Additional file 2.** The ACD-A infusion dose corresponding to blood flow rate in the fixed group

| Blood flow (ml/min) | 110 | 120 | 130 | 140 | 150 | 160 | 170 | 180 |
| --- | --- | --- | --- | --- | --- | --- | --- | --- |
| ACD-A (ml/h) | 234 | 255 | 276 | 297 | 319 | 340 | 361 | 382 |

Abbreviation：ACD-A: anticoagulant citrate dextrose solution-A
