# Supplementary material for: Feasibility and efficacy of modified fixed citrate concentration protocol using only commercial preparations in critically ill patients: a prospective cohort study with a historical control group
Source: BMC Anesthesiol. 2021 Mar 30;21:96. doi: 10.1186/s12871-021-01319-4 (PMC8008573; doi:10.1186/s12871-021-01319-4)
Supplement: Supplementary file 3 — Additional file 3. [file 12871_2021_1319_MOESM3_ESM.docx]

**Additional file 3.** Scheme for the calcium infusion according to effluent flow in the fixed protocol

| Effluent flow (ml/h) | 10% Calcium gluconate (ml/h) | Effluent flow (ml/h) | 10% Calcium gluconate (ml/h) |
| --- | --- | --- | --- |
| 2000 | 7.2 | 3100 | 11.1 |
| 2100 | 7.5 | 3200 | 11.5 |
| 2200 | 7.9 | 3300 | 11.8 |
| 2300 | 8.2 | 3400 | 12.2 |
| 2400 | 8.6 | 3500 | 12.5 |
| 2500 | 9.0 | 3600 | 12.9 |
| 2600 | 9.3 | 3700 | 13.3 |
| 2700 | 9.7 | 3800 | 13.6 |
| 2800 | 10.0 | 3900 | 14.0 |
| 2900 | 10.4 | 4000 | 14.3 |
| 3000 | 10.8 | 4100 | 14.7 |
